# Impact of Enhancer of Zeste Homolog 2 on T Helper Cell-Mediated Allergic Rhinitis

**DOI:** 10.3389/fimmu.2017.00790

**Published:** 2017-07-10

**Authors:** Tsung-Yun Hou, Ming-Rong Chen, Yu-Ching Chou, Po-Chieh Kan, Yi-Ta Tsai, Tai-Lung Cha

**Affiliations:** ^1^Graduate Institute of Medical Sciences, National Defense Medical Center, Taipei, Taiwan; ^2^Division of Rheumatology/Immunology/Allergy, Department of Internal Medicine, Tri-Service General Hospital, National Defense Medical Center, Taipei, Taiwan; ^3^Division of Rheumatology/Immunology/Allergy, Department of Internal Medicine, Wan Fang Hospital, Taipei Medical University, Taipei, Taiwan; ^4^Graduate Institutes of Life Sciences, National Defense Medical Center, Taipei, Taiwan; ^5^School of Public Health, National Defense Medical Center, Taipei, Taiwan; ^6^Division of Urology, Department of Surgery, Tri-Service General Hospital, National Defense Medical Center, Taipei, Taiwan

**Keywords:** allergy, allergy rhinitis, enhancer of zeste homolog 2, T helper cells, epigenetics

## Abstract

Enhancer of zeste homolog 2 (Ezh2) has been shown to play a role in the differentiation of T helper (Th) 1 and 2 cells in mice studies using Ezh2-deficient T cells. However, the results have been inconsistent, and the function of Ezh2 in human Th1 and Th2 cell differentiation and its association with disease remains controversial. We measured the expression of Ezh2 in Th1 and Th2 cells in peripheral blood mononuclear cells after acute challenge with house dust mite using flow cytometry in patients with allergic rhinitis (AR) and controls. The role of Ezh2 was further explored by adding the p38 inhibitor to see if this affected allergen-induced Th1 and Th2 differentiation. The expression of Ezh2 in the Th1 and Th2 cells was significantly lower in the patients than in the controls and was negatively correlated with serum IL-17A levels in the patients. *Ex vivo* allergen challenge resulted in rapid Th2 cell differentiation, which was negatively associated with the Ezh2 expression in Th2 cells. Inhibiting p38 activity increased the expression of Ezh2 in Th2 cells and reduced the number of differentiated Th2 cells. Our findings suggest that Ezh2 expression is potentially associated with AR development.

## Introduction

Allergic rhinitis (AR) is very common worldwide and impacts the ability to work, performance at school, sleep, and quality of life. Genetic and environmental factors are known to affect the development of allergic diseases, and although the incidence of symptomatic allergic diseases varies in different parts of the world, there has been a rise in the prevalence of AR in the last century. T helper (Th) 2 cells are known to play a prominent role in allergic inflammation initiated by an immunological response to allergens such as dust mites. Th2 cells secrete cytokines such as interleukin-4 (IL-4) to induce B cell isotype switching and immunoglobulin E (IgE) secretion (1). On the other hand, various immune mechanisms counterbalance IgE-mediated processes, of which IL-10 has attracted the most attention (1).

During an immune response, antigen-activated naive CD4^+^ T cells can give rise to Th effector subsets that are tailored to their respective roles by secreting specific cytokines. While a growing body of evidence has indicated the complexity of immunological processes in AR, the role of Th2 cells and consequent IgE responses remain at the forefront of current research. Th1 cells produce the signature cytokine IFN-γ, which is a potent antagonist of IL-4, inhibiting the differentiation of Th2 cells (2). An efficient response to foreign antigens results in balance among Th1, Th2, and other Th subsets (2). According to the Th1/Th2 dichotomy (2, 3), each subset orchestrates specific immunological responses to allergens or invasive pathogens, and the concomitant inhibition of the opposing phenotype is equally critical (2). Wong et al. reported that the percentage of IL-4-producing Th2 cells was nearly the same in patients with allergic asthma as in controls, but that the Th1/Th2 cell ratio was lower in the patients (4). This specialization of Th cell subsets is regulated by a network of lineage-specifying transcription factors and epigenetic modifications (5–8), of which epigenetic modifications in Th cell differentiation have attracted particular interest.

Environmental effects on genes are also known to influence the risk of developing allergic diseases and the severity of allergic symptoms through epigenetic processes. An epigenetic modifier of enhancer of zeste homolog 2 (Ezh2), histone methyltransferase, catalyzes the trimethylation of histone H3 at lysine 27 (H3K27me3). Ezh2 is generally considered to be a transcriptional suppressor. O’Carroll et al. reported that Ezh2 was broadly expressed during early mouse embryogenesis, and that the deletion of Ezh2 was embryonically lethal (9). Other murine studies have shown that Ezh2 contributes to Th1 and Th2 cell commitment (6, 10–12); however, the role of Ezh2 still remains unclear. *In vitro* studies have shown that CD4^+^ T cells deficient in Ezh2 secrete increased levels of IFN-γ, IL-4, IL-5, and IL-13 when T cell receptors are activated (5, 10). In addition, Ezh2 has been shown to directly bind to Tbx21 and Gata3 during Th1 and Th2 cell differentiation, suggesting a negative role of Ezh2 in mediating both Th1 and Th2 differentiation (10, 13).

However, Jacob et al. suggested that Ezh2 plays a positive role in mediating both Th1 and Th2 differentiation (14). In addition, an *in vivo* study reported that loss of Ezh2 in CD4^+^ T cells resulted in enhanced allergic inflammation and progressive accumulation of memory phenotype Th2 cells in an ovalbumin-induced model of allergic asthma (10). Moreover, Zhang et al. showed that, as a gene silencer, the loss of Ezh2 in mice decreased the number of Th1 cells when exposed to *Listeria monocytogenes*, even though the expressions of T-bet and IFN-γ increased (11). Furthermore, in a graft-vs.-host disease mice model, the loss of Ezh2 in T cells specifically impaired the differentiation of IFN-γ-producing effector cells, but increased overall production of IL-4 in an alloreactive T cell reaction (6). In a mouse model of human aplastic anemia, Ezh2 was shown to promote the generation of Th1 cells through a mechanism of transcriptional and posttranscriptional regulation of T-bet (12). Taken together, the role of Ezh2 in Th1 and Th2 cells continues to be controversial, and the findings from murine studies cannot be extrapolated to humans. Therefore, due to the lack of human studies and in light of the proposed potential role of Ezh2 in Th1 and Th2 cell fate commitment, we conducted this study. Our results demonstrated that Ezh2 positively regulates Th1 differentiation but negatively regulates Th2 differentiation during an allergic response. We also found that inhibition of p38 induced alterations in the expression of Ezh2 and Th1/2 cell differentiation. These findings may help in the development of new treatments for allergic diseases.

## Materials and Methods

### Ethics and Patient Enrollment

The design of this study with regards to patient enrollment and biological sample collection was approved by the Institutional Review Board of the Ethics Committee on Human Studies of Tri-Service General Hospital, National Defense Medical Center, Taiwan (No. 1-102-05-139 and 2-102-05-025), and the study was conducted in strict accordance with the current Good Clinical Practice and Act of Human Research in Taiwan. All enrollees provided informed consent. A total of 114 allergic and non-allergic patients were enrolled from Tri-Service General Hospital, National Defense Medical Center, Taipei, Taiwan. Eligible AR subjects were 20–75 years of age, with an at least 1-year history of AR.

### Human Peripheral Blood Mononuclear Cell (PBMC) Collection

We measured the expression of Ezh2 in human peripheral Th cells using flow cytometry in both the allergic patients and the non-allergic controls. PBMCs were isolated from peripheral venous blood by Ficoll-Plaque plus (GE Healthcare, Uppsala, Sweden) density gradient centrifugation.

### Human PBMC Analysis

We measured immunoglobulin (Ig) E, white blood cell count, and the percentage of lymphocytes, eosinophils, T cells (CD13), B cells (CD19), Th cells (CD4), T suppressor cells (CD8), NK cells, and naïve T cells in the PBMCs. The percentages of CD3^+^ T cells, B cells, CD4^+^ Th, CD8^+^ suppressor T cells, and NK cells were calculated by flow cytometric analysis using BD Simultest™ IMK-lymphocyte (BD, Catalog No. 340182, San Jose, CA, USA) and BD Simultest IMK-Lymphocyte software. We then calculated the ratio of naïve T cells, activated T cells, Th1 cells, and Th2 cells that were divided by CD4^+^ T cells. We also measured basic variables (as listed in Table 1).

**Table 1:** Demographic data and laboratory characteristics.

Variable	Disease (*n* = 65)	Control (*n* = 49)	*p*-Value^a^

	*N* (%)	*N* (%)	

	Mean ± SE		
Age	35.75 ± 1.42	40.61 ± 2.29	0.074
Sex			0.482
Male	32 (49.23)	20 (40.82)	
Female	33 (50.77)	29 (59.18)	
Immunoglobulin E	790.91 ± 221.15	44.52 ± 6.54	0.001
WBC	7,614.62 ± 306.50	6,909.59 ± 387.49	0.151
Lymphocyte %	28.64 ± 1.05	30.34 ± 1.43	0.327
Eosinophil %	3.28 ± 0.44	2.21 ± 0.32	0.066
T cell (CD3) %	70.62 ± 0.87	69.57 ± 1.40	0.527
B cell (CD19) %	15.89 ± 0.75	16.99 ± 0.99	0.368
T helper (CD4) %	40.58 ± 1.06	38.93 ± 1.17	0.299
T suppressor (CD8) %	25.89 ± 0.93	25.76 ± 1.05	0.925
NK cell %	12.18 ± 0.85	11.93 ± 0.99	0.849
Naïve T/CD4 (%)	56.95 ± 1.93	57.91 ± 1.91	0.730
Activated T/CD4 (%)	43.05 ± 1.93	42.09 ± 1.91	0.730
Th1/CD4 (%)	7.97 ± 0.96	9.54 ± 1.11	0.287
Th2/CD4 (%)	4.64 ± 0.61	4.67 ± 0.66	0.978
**Allergen-specific IgE (kU/l)**			
*Dermatophagoides pteronyssinus*	22.02 ± 3.99	0.79 ± 0.35	<0.001
Cockroach, German (*Blatella germanica*)	1.08 ± 0.19	0.13 ± 0.03	<0.001
Crab	0.86 ± 0.21	0.16 ± 0.07	0.002
Shrimp	1.24 ± 0.26	0.13 ± 0.04	<0.001

*M ± SE, mean ± SE*.

*^a^p-Value by t-test or chi-square*.

### House Dust Mite (HDM) Stimulation

House dust mite extract contains allergens with potent sensitizing abilities in atopic patients. This sensitization to HDM allergens is not only caused by exposure to allergenic compounds of the HDM but also by compounds that facilitate the access of allergens to cells of the immune system. The PBMCs were washed three times and suspended in complete medium RPMI-1640 supplemented with 10% (v/v) fetal calf serum, 2 mM l-glutamine, 1 mM sodium pyruvate, 1 µM 2-mercaptoethanol (Sigma Chemical, Saint-Louis, MO, USA), 1,000 U/ml penicillin and streptomycin. The PBMCs were then cultured in medium containing 10 µg/ml HDM extract with or without 10 ng/ml recombinant human IL-10 (eBioscience) at 37°C in 5% CO2, and the cells were harvested after 0, 5, and 10 days. We evaluated the percentages of Th1 and Th2 cells in the CD4^+^ T cells, and the expression of Ezh2 in the Th1 and Th2 cells compared to the naïve T cells.

### Cell Culture

The human PBMCs were suspended in RPMI-1640 (Gibco) supplemented with 10% fetal bovine serum (Gibco), 50 µg/ml penicillin (Sigma), 50 µg/ml streptomycin (Sigma), and 2 mM l-glutamine (Sigma) and seeded at a concentration of at 1 × 10^6^ cells/ml in six-well plates for proliferation and collection of supernatants at 37°C in a humidified atmosphere of 5% CO_2_. The PBMCs were then cultured in medium containing 10 µg/ml HDM extract with or without p38 MAP kinase inhibitor (10 nM) (Catalog number 506126, Calbiochem Co., La Jolla, CA, USA) at 37°C in 5% CO_2_. The cells were harvested after 0, 5, and 10 days.

Human peripheral T lymphocyte Jurkat cells (American Type Culture Collection) were cultured in RPMI-1640 (Gibco) supplement with 10% fetal bovine serum containing 50 µg/ml penicillin/streptomycin and 2 mM l-glutamate and seeded at a concentration of 1 × 10^5^ cells/ml. The cells were incubated at 37°C in a humidified atmosphere of 5% CO_2_ with or without p38 MAP kinase inhibitor (10 nM) (Catalog number 506126, Calbiochem Co., La Jolla, CA, USA), and then harvested after 0, 2, 4, 6, and 8 days.

### Flow Cytometry Assay

The human PBMCs were at a concentration of 1 × 10^6^ cells/ml in media for 5 h with phorbol 12-myristate 13-acetate/ionomycin (50 ng/ml and 1 µg/ml, respectively) in the presence of BD GolgiStop™ Protein Transport Inhibitor (4 µl of BD GolgiStop™ for every 6 ml of cell culture thoroughly mixed). The cells were then fixed in BD Cytofix™ Fixation Buffer, and permeabilized with BD Phosflow™ Perm/Wash Buffer. Flow cytometric analysis of the expression of Ezh2 was conducted using mouse anti-Ezh2 monoclonal antibodies (BD). The fluorescence histograms were derived from gated events with the forward and side light-scatter characteristics of viable cells. Flow cytometry was performed using a BD FacsCanto™ II Flow Cytometer System. We calculated the mean fluorescence intensity (MFI) of Ezh2 in the Th1 and Th2 cells compared to the MFI of naïve CD4^+^ T cells as: (MFI of the Th cells/MFI of the naïve CD4^+^ T cells) × 100%. Antibodies used in this study described as follows: anti-p38 antibody (ABGENT, Catalog Number: AP3904a, San Diego, CA, USA); anti-pp38 antibody (Thr180/Tyr82, ABGENT, Catalog Number: AP52412, San Diego, CA, USA); anti-Ezh2 antibody (ABGENT, Catalog Number: AW5436-U400, San Diego, CA, USA); anti-IL4 antibody (PE, Clone: 8D4-8, Catalog Number: 12-7049, eBioscience, San Diego, CA, USA); anti-IFN-γ antibody (PE, Clone: 4S B3, Catalog Number: 12-7319, eBioscience, San Diego, CA, USA); anti-CD4 antibody (PERCP-CY5.5, Clone: RPA-T4, Catalog Number: 45-0049, eBioscience, San Diego, CA, USA); anti-CD45ra antibody (APC, Clone: MP4-25D2, Catalog Number: 555490, BD, San Diego, CA, USA); Donkey anti-Rat IgG (H + L) Cross-Adsorbed Secondary Antibody (DyLight^®^488, Catalog Number: SA5-10026, eBioscience, San Diego, CA, USA).

### Western Blot

Cell lysates were collected using RIPA buffer (50 mM Tris–HCl pH = 7.4, 1% NP-40, 0.5% Na-deoxycholate, 0.1% SDS, 150 mM NaCl, 2 mM EDTA, 50 mM NaF, 1 mM phenylmethylsulfonyl fluoride) after stimulation. Protein samples (60 µg) were electrophoresed on 10% SDS polyacrylamide gel and transferred to a nitrocellulose membrane (Bio-Rad). Membrane strips were blocked with 5% non-fat milk in Tris-buffered saline, pH 8.2, containing 0.1% Tween 20, and then incubated overnight at 4°C with a 1:500 dilution of rabbit antibodies against Ezh2, p38, and phosphorylated p38. Blots were incubated and scanned using enhanced chemiluminescence (Thermo Scientific™, Catalog number: 32106). The bands were quantified by densitometry using Media Cybernetics Gel Pro Analyzer software 3.1 (Silver Spring, MD, USA). All sample values were normalized to the control sample group, which was set to 100%.

### Statistical Analysis

All experiments were performed at least three times, and the results are presented as mean ± SEM. All statistical analyses were performed using SPSS software (IBM^®^ SPSS^®^ Statistics 22). Group differences were determined using independent *t*-tests (or the Mann–Whitney *U* test) and chi-square test. Multiple logistic regression was used to estimate odds ratios (ORs) and 95% confidence intervals (CIs) for the association between the expression of Ezh2 and the risk of allergic disease after adjusting for potential risk factors. All statistical tests were two-sided with a significance level of 0.05.

## Results

### Ezh2 Was Involved in the Development of AR

**Figure 1:**
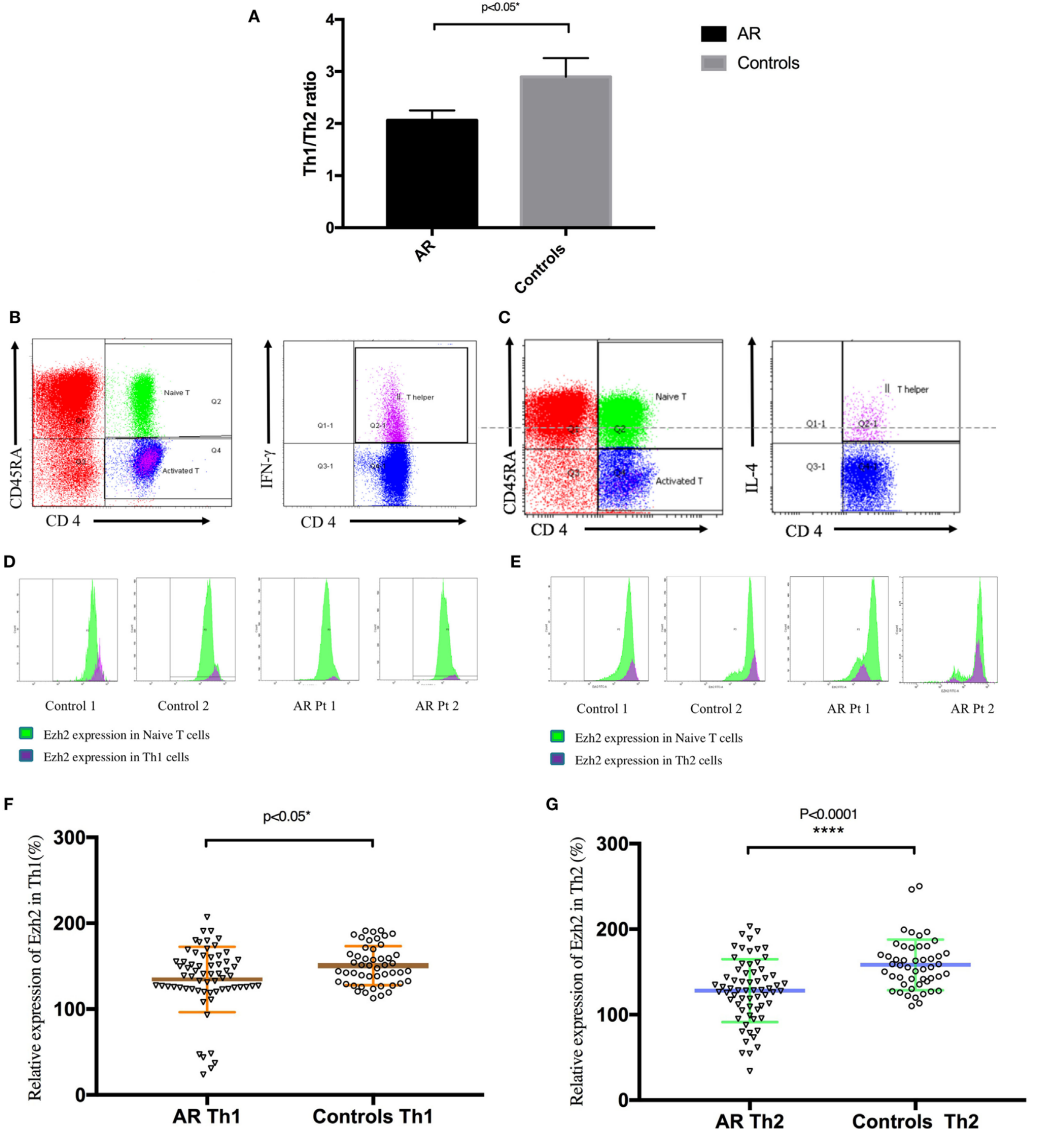

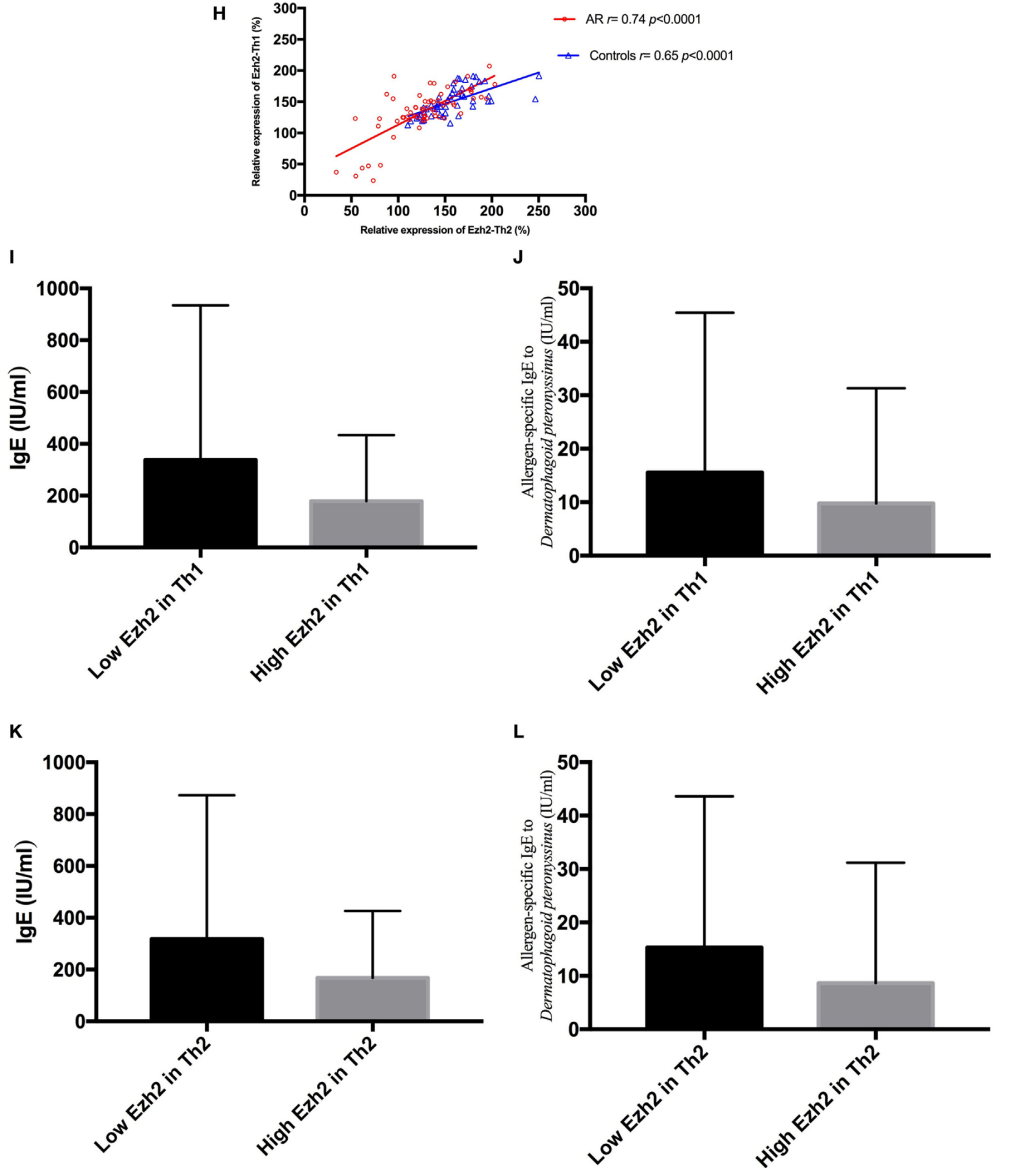
Enhancer of zeste homolog 2 (Ezh2) was involved in the development of allergic rhinitis (AR) in human patients. **(A)** The mean Th1/Th2 cell ratio in the patients with AR and controls. **(B)** Identification of Th1 (CD4^+^CD45RA^−^IFN-γ^+^) cells in peripheral blood mononuclear cells (PBMCs). **(C)** Identification of Th2 (CD4^+^CD45RA^−^IL-4^+^) cells in PBMCs. **(D)** Expression of Ezh2 in Th1 cells in in the patients with AR and controls (two representatives in each group).**(E)** Expression of Ezh2 in Th2 cells in the patients and controls (two representatives in each group). **(F)** Relative Ezh2 expression of Th1 cells to naïve CD4^+^ T cells. The mean fluorescence intensity (MFI) of Th1 cells divided by the MFI of naïve CD4^+^ T cells in the same tube [(MFI of the Th1 cells/MFI of the naïve CD4^+^ T cells) × 100%] (*n* = 65 in the AR group and *n* = 49 in the control group). **(G)** Relative Ezh2 expression of Th2 cells to naïve CD4^+^ T cells. The MFI of Th2 cells divided by the MFI of naïve CD4^+^ T cells in the same tube [(MFI of Th2 cells/MFI of naïve CD4^+^ T cells) × 100%] (*n* = 65 in the AR group and *n* = 49 in the control group). **(H)** Correlation analysis between the relative Ezh2 MFI of Th1 and Th2 cells. **(I)** Total IgE levels in all subjects with lower relative Ezh2 MFI of Th1 cells (lower than the median relative Ezh2 MFI in the controls: 147.60%) compared with those with a lower relative Ezh2 MFI of Th1 cells. **(J)** Allergen-specific IgE to *Dermatophagoides pteronyssinus* levels in all subjects with lower relative Ezh2 MFI of Th1 cells (lower than the median relative Ezh2 MFI in the controls: 147.60%) compared to those with a lower relative Ezh2 MFI of Th1 cells. **(K)** Total IgE levels in all subjects with a lower relative Ezh2 MFI of Th2 cells (lower than the median relative Ezh2 MFI in the controls: 155.52%) compared to those with a lower relative Ezh2 MFI of Th2 cells. **(L)** Allergen-specific IgE to *Dermatophagoides pteronyssinus* levels in all subjects with lower relative Ezh2 MFI of Th2 cells (lower than the median relative Ezh2 MFI in the controls: 155.52%) compared to those with a lower relative Ezh2 MFI of Th2 cells.

We first examined the expression of Ezh2 in human Th1 and Th2 cells from PBMCs from the patients with AR and controls using flow cytometry. The clinical characteristics of the patients (65 cases) and non-allergic controls (49 cases) are summarized in Table 1. There were no significant differences between the two groups in any of the examined variables, although the control group was slightly older than the allergic patients. The mean level of IgE in the patients was significantly higher than that in the controls (790.9 ± 221.15 vs. 44.52 ± 6.54 IU/ml, *p* = 0.001), and the mean percentage (%) of eosinophils was borderline significantly higher in the patients than in the controls (3.28 ± 0.44 vs. 2.21 ± 0.32%, *p* = 0.066). The mean percentage of Th1 cells in the CD4^+^ T cells was lower in the patients than in the controls (7.97 ± 0.9 vs. 9.54 ± 1.1%, *p* = *0.287*), although the difference was not statistically significant. Even though the mean percentages of Th2 cells in the CD4^+^ T cells in the patients and controls were almost the same (4.6 ± 0.61 vs. 4.6 ± 0.66%, *p* = 0.978), the Th1/Th2 cell ratio was significantly lower in the patients than in the controls (2.06 ± 0.19 vs. 2.90 ± 0.36, *p* < 0.05, Figure 1A).

Although the mean percentages of Th2 cells in CD4^+^ T cells in the patients and controls were almost the same, there were significant differences in symptoms, total IgE, and allergen-specific IgE to *Dermatophagoides pteronyssinus*, German cockroach, crab, and shrimp (Table 1). We assumed that these differences were not due to the percentage of Th2 cells, but because of Th2 functional alterations mediated by an epigenetic modifier such as Ezh2. We then measured the expression of Ezh2 in the Th1 and Th2 cells in the PBMCs. Detection of the expression of Ezh2 on the flow cytometer was based on three elements. CD45RA is expressed on naïve T cells, and T cells lose their expression of CD45RA after exposure to antigens. First, naïve CD4^+^ T cells were identified by positive staining for CD45RA and CD4. Next, Th1 (CD4^+^CD45RA^−^IFN-γ^+^) cells and Th2 (CD4^+^CD45RA^−^IL-4^+^) cells were identified by intracellular IFN-γ or interleukin-4 (IL-4) staining (Figures 1B,C). Th1 cells selectively produce IFN-γ and Th2 cells selectively produce IL-4. The expression of Ezh2 was then detected using Ezh2 antibodies from naïve CD4^+^ T cells, gated Th1 (CD4^+^CD45RA^−^IFN-γ^+^) cells and Th2 (CD4^+^CD45RA^−^IL-4^+^) cells (Figures 1D,E). The expression of Ezh2 in Th2 cells was notably decreased in the patients compared to the controls (Figure 1E). We then examined the MFI of Ezh2 in Th1 and Th2 cells in the PBMCs by flow cytometry. The relative MFI of Ezh2 was used to correct for batch-to-batch and person-to-person instrumental variations in fluorescence intensity measurements as follows: (MFI of Th1 or Th2 cells/MFI of naïve CD4^+^ T cells) × 100%. The relative MFI of Ezh2 in the Th1 and Th2 cells was significantly lower in the patients than in the controls (Figures 1E,F).

The relative Ezh2 MFI of Th1 cells was lower in the patients than in the controls (134.59 ± 4.73 vs. 150.66 ± 3.26%, *p* = 0.010, Figure 1F), and the mean Ezh2 MFI of Th2 cells was also significantly lower in the patients than in the controls (128.11 ± 4.55 vs. 158.28 ± 4.23%, *p* < 0.001, Figure 1G). Correlation analysis between the mean Ezh2 MFI of Th1 and Th2 cells showed a strong correlation in both patients and controls (*r* = 0.74, *p* ≤ 0.0001; *r* = 0.65, *p* ≤ 0.0001; respectively, Figure 1H), suggesting that concurrent changes in the expressions of Ezh2 in Th1 and Th2 cells may be linked to Th1/2 polarity. Both higher levels of total IgE and allergen-specific IgE to *Dermatophagoides pteronyssinus* were found, but without statistical significance, in those with a lower relative Ezh2 MFI of Th1 cells (lower than the median relative Ezh2 MFI of the controls of 147.60%) (*p* = 0.083 and *p* = 0.248, respectively, Figures 1I,J) and with lower relative Ezh2 MFI of Th2 cells (lower than the median relative Ezh2 MFI of the controls of 155.52%) (*p* = 0.123 and *p* = 0.208, respectively, Figures 1K,L). These results suggested that Ezh2 plays an important role in Th1 and Th2 cell commitment and the development of allergy in humans.

Table 2 shows the ORs of the risk of AR associated with the expression of Ezh2. The OR for having a relative Ezh2 MFI of Th2 cells lower than the median of the controls (relative Ezh2 MFI: 155.52%) was 3.80 (95% CI: 1.68–8.57). Therefore, a lower expression of Ezh2 in Th2 cells suggested a higher risk of AR. After adjusting for potential confounders including age at enrollment, IgE, eosinophils, allergen-specific IgE to *Dermatophagoides pteronyssinus*, German cockroach, crab, and shrimp, the ORs consistently showed a significant inverse relationship in the incidence rate of AR across Ezh2 MFI Th2/naïve T cell ratio [adjusted OR: 11.94 (95% CI: 2.22–64.39), Table 3].

**Table 2:** Odds ratios of allergic rhinitis related to enhancer of zeste homolog 2 (Ezh2) expression.

Variable^a^^,^^b^	Disease (*n* = 65)	Control (*n* = 49)	Odds ratio (95% CI)

	*N* (%)	*N* (%)	
**Ezh2 MFI Th1/naïve (%)**			
≤147.60	39 (60.00)	24 (48.98)	1.56 (0.74–3.30)
>147.60	26 (40.00)	25 (51.02)	1.00 (reference)
**Ezh2 MFI Th2/naïve (%)**			
≤155.52	51 (78.46)	24 (48.98)	3.80 (1.68–8.57)
>155.52	14 (21.54)	25 (51.02)	1.00 (reference)

*^a^The total number of cases and controls does not correspond because of missing data*.

*^b^Variables were categorized based on the median distribution among control subjects*.

*CI, confidence interval*.

**Table 3:** Adjusted odds ratios of allergic rhinitis related to enhancer of zeste homolog 2 (Ezh2) expression.

	Model 1	Model 2	Model 3
	
Variable^a^^,^^b^	Odds ratio (95% CI)	Odds ratio (95% CI)	Odds ratio (95% CI)
**Ezh2 MFI Th1/naïve (%)**			
≤147.60	1.58 (0.74–3.38)	1.55 (0.56–4.28)	2.45 (0.71–8.47)
>147.60	1.00 (reference)	1.00 (reference)	1.00 (reference)
**Ezh2 MFI Th2/naïve (%)**			
≤155.52	3.57 (1.56–8.13)	8.40 (2.13–33.17)	11.94 (2.22–64.39)
>155.52	1.00 (reference)	1.00 (reference)	1.00 (reference)

*^a^The total number of cases and controls does not correspond because of missing data*.

*^b^Variables were categorized based on the median distribution among control subjects*.

*CI, confidence interval*.

*Model 1: adjustment for age at enrollment*.

*Model 2: adjustment for age at enrollment, immunoglobulin E, eosinophil*.

*Model 3: adjustment for age at enrollment, immunoglobulin E, eosinophil, allergen-specific IgE to Dermatophagoides pteronyssinus, cockroach German, crab, and shrimp*.

**Figure 2:**
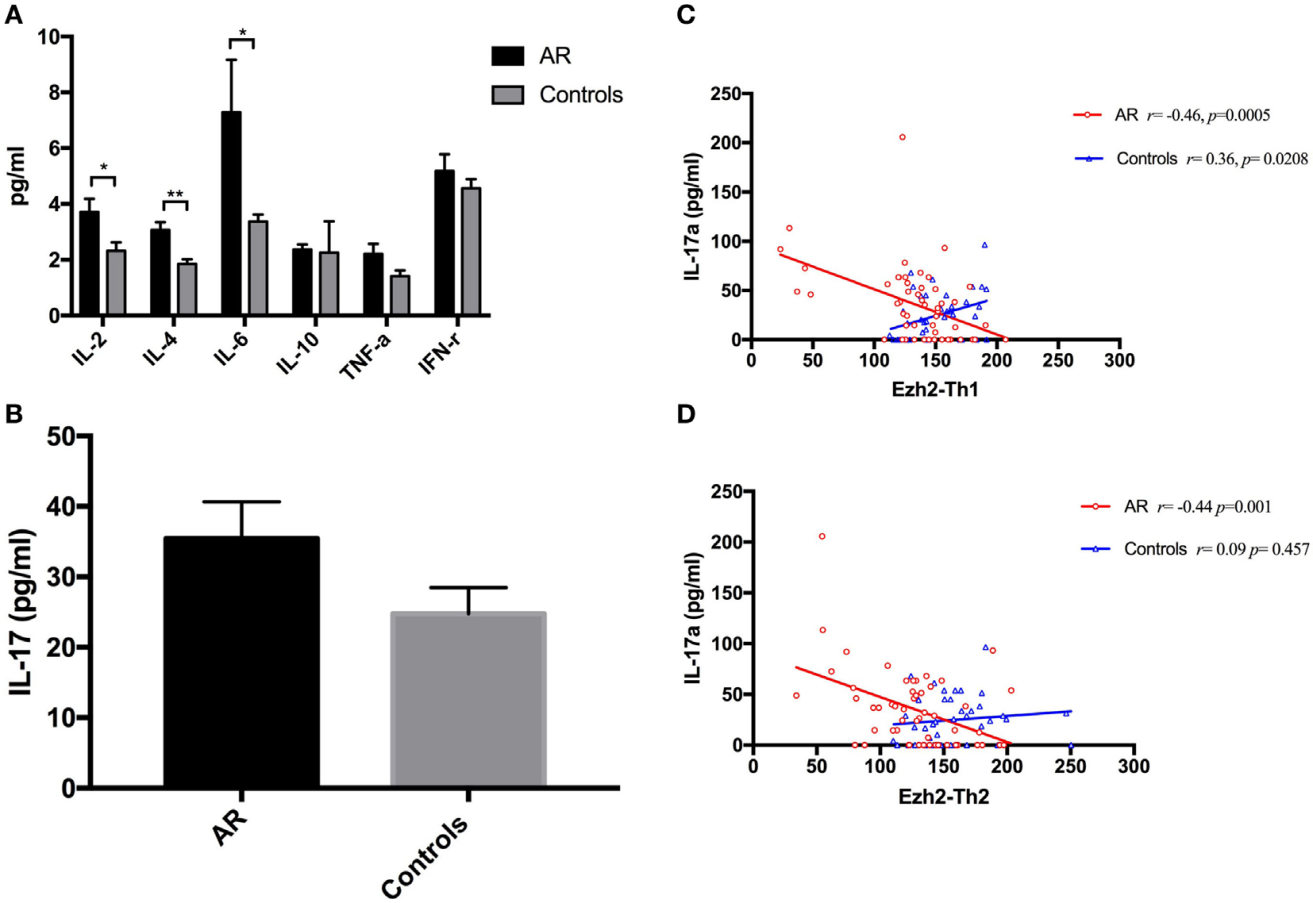
Mean serum cytokine levels and correlation analysis to enhancer of zeste homolog 2 (Ezh2). **(A)** Mean serum cytokine levels including IL-2, interleukin-4, IL-6, IL-10, TNF-α, and IFN-γ in the patients with allergic rhinitis and controls. **(B)** Mean serum IL-17A level in the patients and controls. **(C)** Correlation analysis between the relative Ezh2 expression in Th1 cells with IL-17A in the patients and controls. **(D)** Correlation analysis between the relative Ezh2 expression in Th2 cells with IL-17A in the patients and controls.

To better understand how epigenetic modifiers mediate the balance of Th1 and Th2 immune responses and related cytokine concentrations, we measured fresh-isolated serum cytokine levels using a BD™ cytometric bead array human Th1/Th2/Th17 kit. The patients with AR had a significantly higher levels of serum IL-4 (3.07 ± 0.28 vs. 1.85 ± 0.17 pg/ml, *p* = 0.001), IL-2 (3.71 ± 0.48 vs. 2.32 ± 0.31 pg/ml, *p* = 0.016), and IL-6 (7.29 ± 1.88 vs. 3.37 ± 0.25 pg/ml, *p* = 0.043, Figure 2A) compared to the controls. The mean level of TNF-α in the patients was borderline significantly higher than in the controls (2.21 ± 0.36 vs. 1.41 ± 0.21 pg/ml, *p* = 0.060). No significant differences in IL-10 and IFN-γ levels were observed between the two groups. The mean level of IL-17A in the patients was borderline significantly higher than in the controls (*p* = 0.099, Figure 2B). We then performed correlation analysis between the mean relative expressions of Ezh2 in the Th1 and Th2 cells with cytokine levels, which revealed significant correlations between the mean Ezh2 MFI of Th1 cells with serum IL-17 levels in the patients and controls (*r* = −0.46, *p* = 0.0005 vs. *r* = 0.36, *p* = 0.0208, respectively, Figure 2C). The mean Ezh2 MFI Th2/naïve T cell ratio was significantly negatively correlated with serum IL-17 levels in the patients (*r* = −0.44, *p* = 0.001, Figure 2D), but not in the controls.

### Ezh2 Mediated the *Ex Vivo* HDM-Induced T-lymphocyte Response

**Figure 3:**
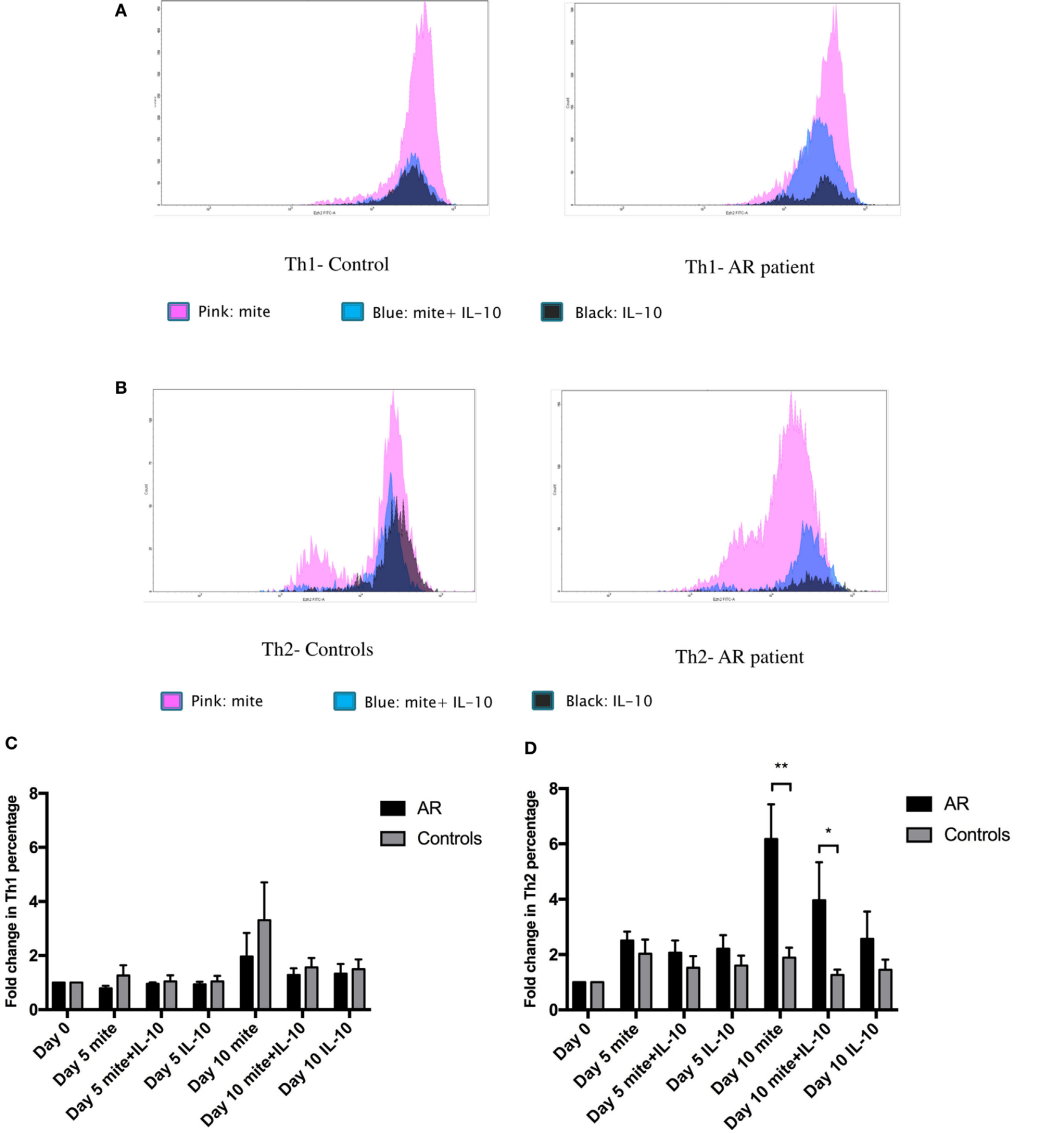

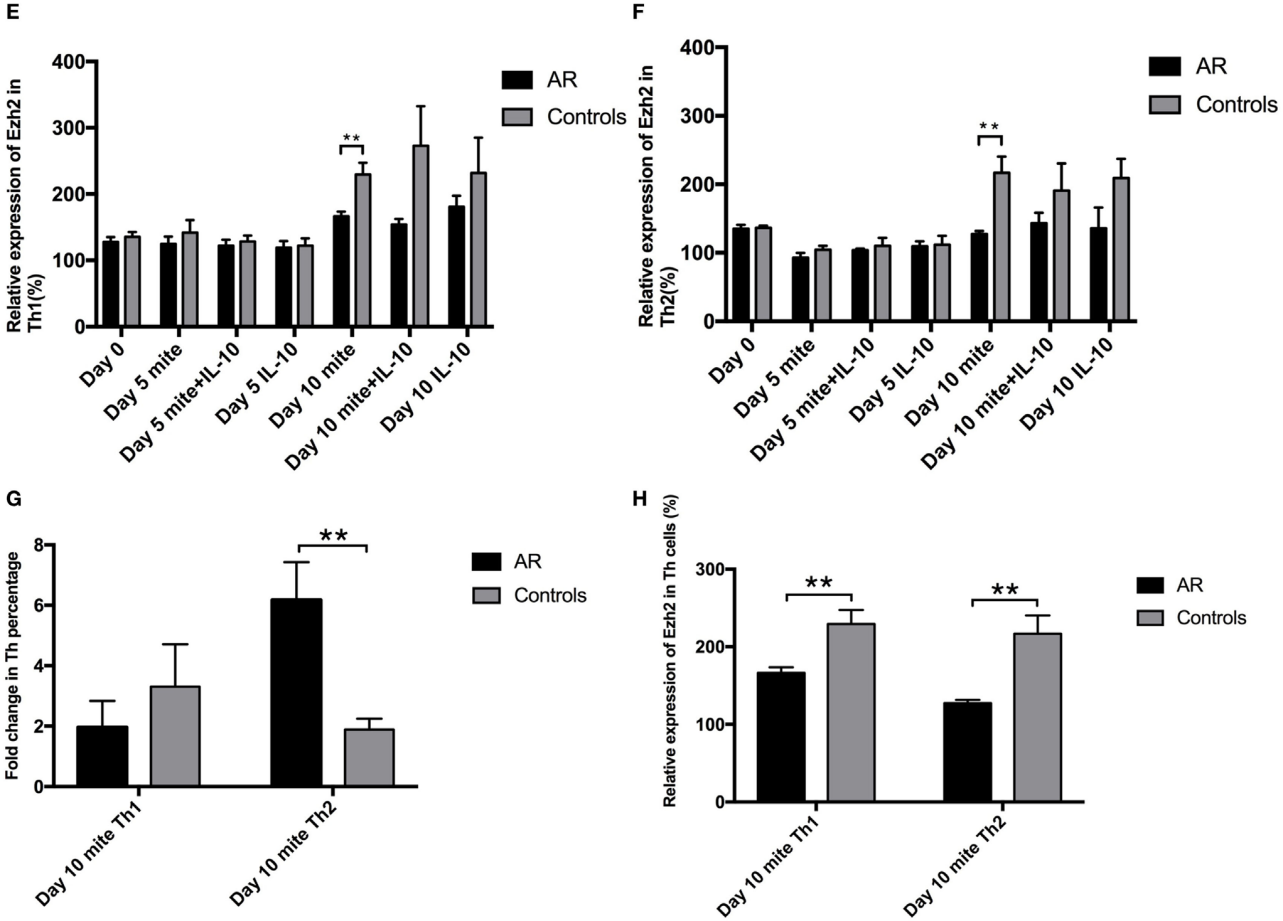
Enhancer of zeste homolog 2 (Ezh2) mediated the *ex vivo* house dust mite (HDM)-induced T-lymphocyte response. **(A)** Histogram showing Ezh2 expression in Th1 cells in one patient with allergic rhinitis and a healthy control by flow cytometric analysis. Peripheral blood mononuclear cells (PBMCs) were cultured in medium containing 10 µg/ml HDM extract (purple color), 10 µg/ml HDM extract, and recombinant human IL-10 (10 ng/mL) (light blue color) or recombinant human IL-10 (10 ng/mL) (green color). **(B)** Histogram showing Ezh2 expression in Th2 cells in one patient and a healthy control by flow cytometric analysis. PBMCs were cultured in medium containing 10 µg/ml HDM extract (purple color), 10 µg/ml HDM extract, and recombinant human IL-10 (10 ng/mL) (blue color) or recombinant human IL-10 (10 ng/mL) (green color). **(C)** The mean fold change of Th1 percentage on day 10 compared to day 0 (the percentage on day 0/the percentage on day 10) (*n* = 5 in each group). **(D)** The mean fold change of Th2 percentage on day 10 compared to day 0 (the percentage on day 0/the percentage on day 10) (*n* = 5), *p < 0.05, **p < 0.01, by the Mann–Whitney *U* test. **(E)** Relative Ezh2 expression of Th1 cells to naïve CD4^+^ T cells. The mean fluorescence intensity (MFI) of Th1 cells divided by the MFI of naïve CD4^+^ T cells in the same tube [(MFI of Th1 cells/MFI of naïve CD4^+^ T cells) × 100%] (*n* = 5 in each group). **(F)** Relative Ezh2 expression of Th2 cells to naïve CD4^+^ T cells. The MFI of Th2 cells divided by the MFI of naïve CD4^+^ T cells in the same tube [(MFI of Th2 cells/MFI of naïve CD4^+^ T cells) × 100%] (*n* = 5 in each group). **(G)** The mean fold change of Th1 and Th2 percentages after 10 days of HDM stimulation compared to day 0 (the percentage on day 0/the percentage on day 10) (*n* = 5 in each group), **p < 0.01, by the Mann–Whitney *U* test. **(H)** Relative Ezh2 expression of Th1 and Th2 cells to naïve CD4^+^ T cells after 10 days of HDM stimulation (*n* = 5 in each group), **p < 0.01, by the Mann–Whitney *U* test.

House dust mite is the most common allergen among patients with AR. The prevalence rates of *Dermatophagoides pteronyssinus* and *Dermatophagoides farinae* in Taiwan have been reported to be 87.5 and 82.1%, respectively (15, 16). HDM allergens play a crucial role in the development of AR and asthma, and its allergenic effects are thought to be orchestrated through the CD4^+^ Th2 cells that drive the IgE-dependent allergic response (15). The level of Th cells in patients allergic to HDM when stimulated with the HDM allergen has been reported to be significantly different from controls (17, 18). Similarly, the responsiveness of T lymphocytes to allergen challenge *in vitro* has been reported to play a role in determining the allergic response to the allergen *in vivo* (18). In order to determine whether Ezh2 influences the T lymphocyte response to HDM challenge, we measured the percentage of Th1 and Th2 cells in CD4^+^ T cells, and the associated expression of Ezh2 using flow cytometry. The expression of Ezh2 in Th1 cells after 10 days of HDM allergen stimulation is shown in Figure 3A. There was no obvious difference in Ezh2 expression between the patients and controls. However, after adding IL-10, the expression of Ezh2 in the Th1 cells decreased (Figure 3A). With regards to Th2 cells, the patients had a significantly higher number of Th2 cells and a significantly lower expression of Ezh2 in the Th2 cells compared to the controls after 10 days of HDM allergen stimulation (Figure 3B).

We then analyzed the mean fold increase in Th1 and Th2 cells on day 10 compared to day 0 (the percentage on day 0/the percentage on day 10) to avoid individual differences. After 10 days of stimulation with the HDM allergen, the PBMCs from the patients had a smaller fold increase in Th1 cells than the controls (1.96 ± 0.88 vs. 3.31 ± 1.40, *p* = 0.465, Figure 3C), but a significantly higher fold increase in Th2 cells than the controls (6.18 ± 1.25 vs. 1.89 ± 0.36, *p* = 0.009, Figure 3D). The effect of HDM-induced responses was inhibited by adding IL-10 (Figures 3C,D).

In our *ex vivo* study, the PBMCs from the patients had a smaller decrease in Th1 cells but a significant increase in Th2 cells in CD4^+^ T cells compared to the controls after stimulation with HDM allergen for 10 days (Figure 3G). In addition, there were significant decreases in Ezh2 expression in both Th1 and Th2 cells compared with the controls (Figures 3E,F,H).

### HDM-Induced Ezh2-Mediated T-lymphocyte Response Was Modified by the p38 Inhibitor

**Figure 4:**
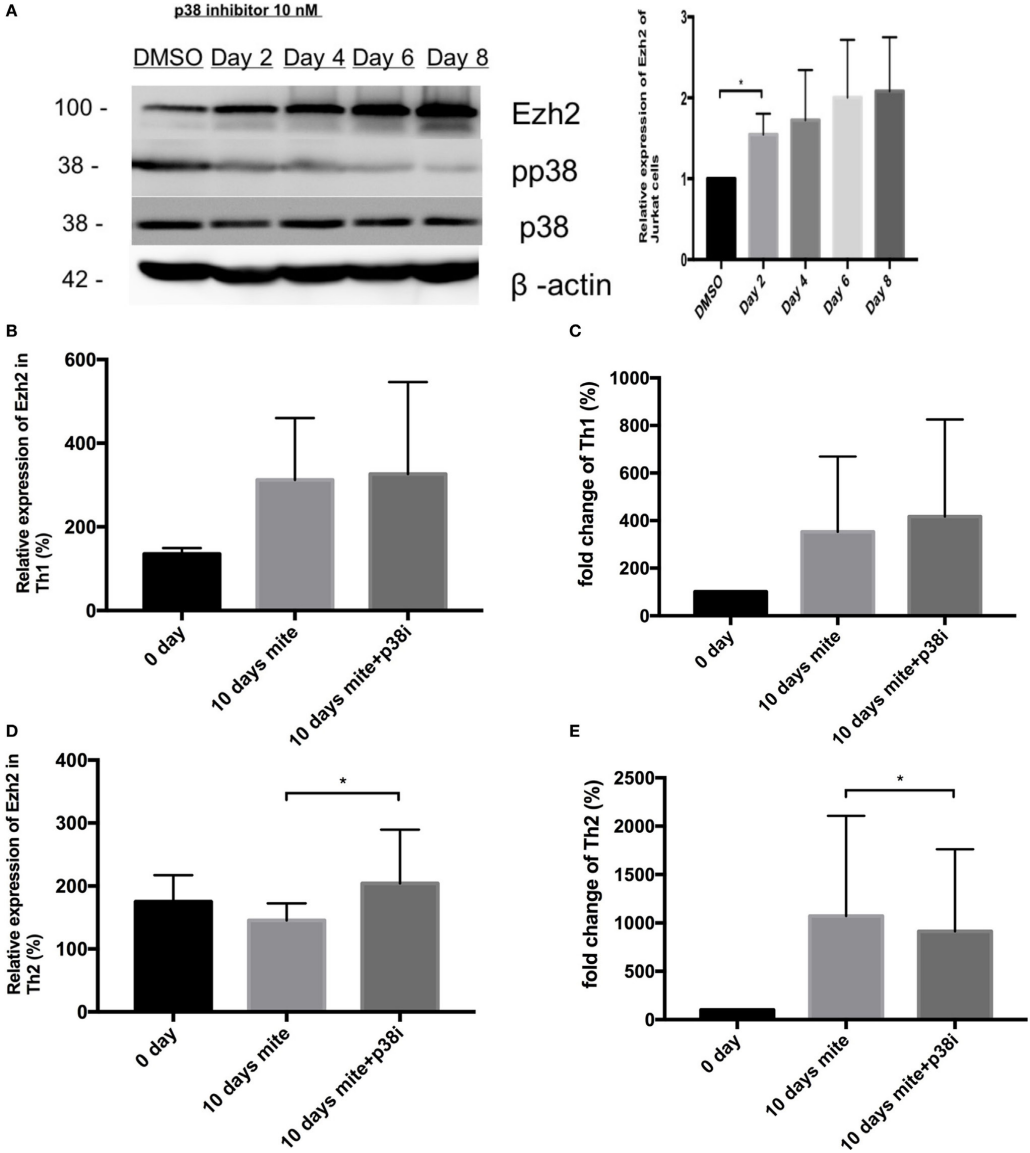
House dust mite-induced enhancer of zeste homolog 2 (Ezh2)-mediated T-lymphocyte response was modified by p38 inhibitor. **(A)** Western blot analysis of Ezh2, p38, pp38, and Actin in Jurkat cells after adding p38 MAP kinase inhibitor, *p < 0.05, by the *t*-test. **(B)** Relative Ezh2 expression of Th1 cells to naïve CD4^+^ T cells with HDM stimulation with or without p38 MAP kinase inhibitor treatment on day 10. The mean fluorescence intensity (MFI) of Th1 cells divided by the MFI of naïve CD4^+^ T cells in the same tube [(MFI of Th1 cells/MFI of naïve CD4^+^ T cells) × 100%] (*n* = 11). **(C)** The mean fold change of Th1 percentage in CD4^+^ T cells with HDM stimulation with or without p38 MAP kinase inhibitor treatment on day 10 compared to day 0 (the percentage on day 0/the percentage on day 10) (*n* = 11). **(D)** Relative Ezh2 expression of Th2 cells to naïve CD4^+^ T cells with HDM stimulation with or without p38 MAP kinase inhibitor treatment on day 10. The MFI of Th1 cells divided by the MFI of naïve CD4^+^ T cells in the same tube [(MFI of Th2 cells/MFI of naïve CD4^+^ T cells) × 100%] (*n* = 11). **(E)** The mean fold change of Th2 percentage in CD4^+^ T cells with HDM stimulation with or without p38 MAP kinase inhibitor treatment on day 10 compared to day 0 (the percentage on day 0/the percentage on day 10) (*n* = 11 in each group).

Recent studies have shown that p38 directly downregulates the expression of Ezh2, and the p38 inhibitor has emerged as a novel regulator of Ezh2 (19). In previous studies using Th2 cells, p38 has been shown to strongly inhibit IL-4 production (20). The anti-allergic activity of p38 has also been demonstrated by essential abolition (~93% inhibition) in inhaled ovalbumin-induced airway eosinophilia in mice. In addition, studies using ovalbumin-induced, Sephadex-induced airway inflammation of rats or inhaled ovalbumin-induced airway eosinophilia also support the potential utility of p38 kinase inhibitors for the treatment of allergic inflammatory disorders (21–23). Therefore, we hypothesized that the inhibitory effect of p38 resulting in Ezh2 upregulation would affect allergen-induced Th1 and Th2 differentiation. To test this hypothesis, we first exposed Jurkat cells, a T-cell leukemia line, to the p38 MAP kinase inhibitor (10 nM) (Catalog number 506126, Calbiochem Co., La Jolla, CA, USA), which resulted in a decrease in the level of p-p38 and a significant increase in the level of Ezh2 (Figure 4A). This is consistent with previous studies in which p38 MAPK signaling was shown to negatively regulate the expression of Ezh2. In *ex vivo* HDM stimulation, the relative expression of Ezh2 in Th1 cells increased slightly after adding the p38 MAP kinase inhibitor (312.10 ± 148.39 vs. 326.25 ± 220.20%, *p* = 0.72, Figure 4B), with a borderline increase in fold change of Th1 cells (352.86 ± 316.76 vs. 417.17 ± 408.50%, *p* = 0.083, Figure 4C). In comparison, the relative expression of Ezh2 in Th2 cells significantly increased after adding the p38 MAP kinase inhibitor (145.55 ± 26.88 vs. 204.08 ± 85.32%, *p* = 0.029, Figure 4D), but with a decrease in fold change of Th2 cells (1,071.10 ± 1,037.04 vs. 913.78 ± 847.95%, *p* = 0.035, Figure 4E). The addition of the p38 MAP kinase inhibitor resulted in a trend of an increase in Th1 cells and a decrease in Th2 cells.

## Discussion

CD4^+^ Th lymphocytes play a major role in the development of allergic disorders, and it is generally accepted that an imbalance in the different subsets of allergen-specific CD4^+^ T cells may result in the development of allergic diseases. In contrast to non-allergic individuals, those who are allergic show an aberrant Th2-dominated response to allergens due to ineffective counter regulation by allergen-specific Th1 and Treg cells with the signature IL-10 cytokine (1). Ezh2 is known as the functional enzymatic component of Polycomb Repressive Complex 2, and it regulates embryonic development and cell differentiation through the epigenetic regulation of genes responsible for them (24). Several murine studies have reported that Ezh2 contributes to Th1 and Th2 cell commitment; however, how Ezh2 affects Th1 and Th2 cells is still controversial. In particular, whether and how Ezh2 is involved in human Th cell differentiation remains unclear.

In the current human study, the patients with AR had similar percentages of total T cells and CD4^+^ Th cells as the controls. In addition, the percentage of Th2 cells in the CD4^+^ T cells was almost equivalent in both groups; however, significant functional dissimilarities including a prominent level of IgE, Th2 pattern of cytokine levels, and eosinophilia were noted in the patients. We used flow cytometry to evaluate the expression of Ezh2 in PBMCs, which is one of the most powerful tools for single-cell analysis of the immune system at a cellular level. However, it is limited by the lack of standardization of individual variability to fluorescence staining (25). For example, in a study on intracellular cytokine staining standardization involving 15 institutions, the mean inter-laboratory coefficient of variation ranged from 17 to 44%, even though the cell preparation was standardized and the testing was performed using the same samples and reagents at each site (25, 26). The MFI ratio has been used in previous studies (27, 28). We used the MFI ratio of the relative MFI of Ezh2 to avoid batch-to-batch and person-to-person instrumental variations in fluorescence intensity. The relative MFI of Ezh2 is the mean MFI of Th1 or Th2 cells divided by the MFI of naïve CD4^+^ T cells in the same tube [(MFI of Th1 or Th2 cells/MFI of naïve CD4^+^ T cells) × 100%]. The aim of using the ratio of upstream naïve CD4^+^ T cells and the hierarchical downstream populations of Th1 or Th2 in the same tube was to avoid bias in detecting the Ezh2 expression in human PBMCs.

The expressions of Ezh2 in Th1 and Th2 cells in the PBMCs from our patients with AR were lower than those in the non-allergic controls. In addition, a higher risk of AR was associated with a lower Ezh2 expression in Th2 cells (OR 3.80, 95% CI 1.68–8.57). Furthermore, the lower Ezh2 expression in Th2 cells was independently associated with a very high risk of AR (OR 11.94, 95% CI 2.22–64.39) after adjusting for potential confounding factors. Therefore, the ratio of the expression of Ezh2 in Th2 cells to naïve CD4^+^ T cells may be used to predict the risk of AR and other allergic disorders even before obvious symptoms occur.

The expression of Ezh2 in Th1 and Th2 cells was also positively highly correlated in both the patients and controls (Figure 1H). Combined with our *ex vivo* results (Figures 3G,H), Ezh2 seems to play a negative role in mediating the differentiation of Th2 cells. This finding clarifies the inconsistent results reported in previous murine studies (10–12). Ezh2 seems to be involved in a counter-regulatory mechanism of the Th1/Th2 dichotomy, allowing for the stable expression of the Th2 cell phenotype.

Enhancer of zeste homolog 2 is a chromatin-modifying enzyme involved in silencing gene expression by substantial trimethylation at lysine 27 of histone 3 (H3K27me3) and direct binding to Gata3 in Th2 cells, and the conditional deletion of Ezh2 has been shown to decrease H3K27me3 binding over IL-4 and IL-5 promotor sites in mice (10). Moreover, Ezh2 deficiency in mice has been shown to result in the spontaneous generation of discrete Th2 cytokine-producing populations (10). Our results using human PBMCs showed a lower expression of Ezh2 in the Th2 cells of the patients with AR, suggesting that less gene repression can facilitate Th2 cell differentiation, which is consistent with a previous *in vivo* mice study where the loss of Ezh2 caused accumulation of memory phenotype Th2 cells that mediated the development of allergic asthma (10). In this study, the expression of Ezh2 in Th1 cells in the patients with AR seemed to have a positive influence on Th1 cell differentiation. Although silencing of the IFN-γ loci in naive CD4^+^ T cells is dependent on Ezh2, the positive influence of Ezh2 on Th1 cells differs from its usual gene repression effect. Several explanations have been proposed in earlier mice studies, including the loss of Ezh2 specifically impairing the differentiation of Th1 cells through a mechanism of transcriptional and posttranscriptional regulation of T-bet (6, 12).

The differentiation and activation functions of cytokines regulate allergic responses. IL-17A has a proallergic effect, and elevated levels of IL-17A have been reported in allergic patients (4, 29). A higher level of IL-17-producing Th2 cells has also been reported in patients with atopic asthma and a mouse model of allergic lung diseases (30). Th2/Th17 predominant (IL-4/IL-17 dual-positive Th cells) cells have been reported in patients with severe asthma (31). IL-17 production in dual-positive cells has been associated with IL4^high^ CD4 T cells (31), and Th2 cells can further differentiate into dual-positive Th2/Th17 cells, so that Th2 cells are the favored precursors for Th2/Th17 cells (31). Our results showed higher IL-17A levels in the patients compared to the controls, which is consistent with a previous study (4). The expressions of Ezh2 in Th1 and Th2 cells were negatively correlated with serum IL-17 levels in our patients, but positively correlated in the controls (Figures 2C,D). This difference may indicate a higher risk of developing an allergic disease. Our results provide epigenetic evidence of an association between Th2 cells and IL-17, and this association may be through histone modification.

There are several limitations to this study. First, we only evaluated the overall expression of Ezh2, but did not establish gene-specific epigenetic profiles relating to the expression of Ezh2 in the patients with AR. The underlying enzymatic mechanism remains unclear, especially with regards to different human genes. In addition, the reported variations in the expression of Ezh2 are functionally relevant to the severity of symptoms, and subsequent modification of the allergic phenotype has yet to be clarified. Second, Lentivirus and retroviruse have been used to integrate transgenic constructs into primary T cells, but these procedures cannot site-specifically disrupt or insert genetic elements. In addition, some approach such as Ezh2 knockdown or rescue studies is not obtainable due to a limited lifespan and editing efficiency of primary human T cells.

**Figure 5:**
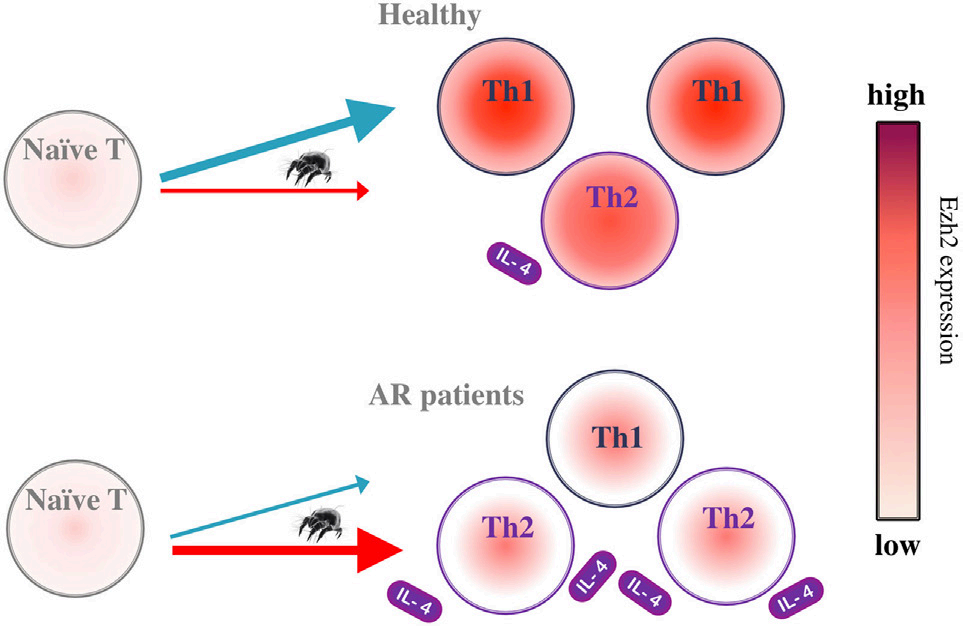
Schematic representation of the proposed enhancer of zeste homolog 2-dependent Th1 and Th2 cell regulation in HDM-induced allergic rhinitis.

In summary, the results from our human PBMCs demonstrated the expressions of Ezh2 in Th1 and Th2 cells were lower in the patients than in the controls. In addition, the expressions of Ezh2 in Th1 and Th2 cells were relatively lower after HDM stimulation in the patients compared to the controls (Figure 5). Combined with previous conditional deletion of Ezh2 mice studies (10, 11), Ezh2 negatively regulates Th2 differentiation. Many publications have addressed the role of epigenetic regulation in allergic diseases and how environmental exposure such as to HDM may induce these molecular events (32). Histone modifications is one of the most common epigenetic mechanisms, and histone modification in T cells plays an important role in the differentiation of T cells toward different T cell subsets, thereby playing a pivotal role in both physiological and pathological conditions including allergy (32). Epigenetic modifications and novel approaches to their regulation may allow for the development of new drugs, as these modifications are potentially reversible. Epigenetic regulation by Ezh2 may therefore be a potential target for Th cell-related pharmacological therapy. Because of the interdependence of epigenetic regulation, combinations of traditional therapy with targeted Ezh2 approaches may have greatest clinical efficacy. Furthermore, the epigenetic regulation of Th cells by Ezh2 may expand our horizon in pharmacological therapy for imbalanced Th1- or Th2-associated inflammatory diseases.

## Ethics Statement

This study with regards to patient enrollment and biological sample collection was approved by the Institutional Review Board of the Ethics Committee on Human Studies of Tri-Service General Hospital, National Defense Medical Center, Taiwan (No. 1-102-05-139 and 2-102-05-025), and the study was conducted in strict accordance with the current Good Clinical Practice and Act of Human Research in Taiwan. All subjects gave written informed consent in accordance with the Declaration of Helsinki.

## Author Contributions

T-YH, M-RC, and T-LC designed experiment. T-YH, M-RC, Y-TT, and P-CK performed experiments. T-YH, M-RC, Y-CC, and T-LC wrote the manuscript.

## Conflict of Interest Statement

The authors declare that the research was conducted in the absence of any commercial or financial relationships that could be construed as a potential conflict of interest.
